# Antimicrobial Peptide Dendrimers and Quorum-Sensing Inhibitors in Formulating Next-Generation Anti-Infection Cell Therapy Dressings for Burns

**DOI:** 10.3390/molecules26133839

**Published:** 2021-06-24

**Authors:** Paris Jafari, Alexandre Luscher, Thissa Siriwardena, Murielle Michetti, Yok-Ai Que, Laurence G. Rahme, Jean-Louis Reymond, Wassim Raffoul, Christian Van Delden, Lee Ann Applegate, Thilo Köhler

**Affiliations:** 1 Regenerative Therapy Unit (UTR), Department of Musculoskeletal Medicine DAL, Lausanne University Hospital, 1011 Lausanne, Switzerland; Paris.jafari@utah.edu (P.J.); Murielle.Michetti@chuv.ch (M.M.); 2 Service of Plastic, Reconstructive & Hand Surgery, Lausanne University Hospital, 1011 Lausanne, Switzerland; Wassim.Raffoul@chuv.ch; 3 Department of Pharmaceutics and Pharmaceutical Chemistry, College of Pharmacy, University of Utah, Salt Lake City, UT 84112, USA; 4 Department of Microbiology and Molecular Medicine, University of Geneva, 1211 Geneva, Switzerland; Alexandre.Luscher@unige.ch (A.L.); christian.vandelden@hcuge.ch (C.V.D.); 5 Department of Chemistry, Biochemistry and Pharmaceutical Sciences, University of Bern, 3012 Bern, Switzerland; Thissa.siriwardena@dcb.unibe.ch (T.S.); jean-louis.reymond@dcb.unibe.ch (J.-L.R.); 6 Department of Intensive Care Medicine, Inselspital, Bern University Hospital, University of Bern, 3010 Bern, Switzerland; yok-ai.que@insel.ch; 7 Department of Surgery, Harvard Medical School and Massachusetts General Hospital, Boston, MA 02114, USA; LGRAHME@PARTNERS.ORG; 8 Shriners Hospitals for Children Boston, Boston, MA 02114, USA; 9 Department of Microbiology and Immunobiology, Harvard Medical School, Boston, MA 02115, USA; 10 Division on Infectious Disease and Transplantation, University Hospital of Geneva, 1205 Geneva, Switzerland; 11 Center for Applied Biotechnology and Molecular Medicine, University of Zurich, Winterthurerstrasse 260, 8057 Zurich, Switzerland; 12 Oxford OSCAR Suzhou Center, Oxford University, Suzhou 215028, China

**Keywords:** antimicrobial peptide dendrimers, quorum-sensing inhibitors, MvfR, PqsR, anti-infection dressing, cell therapy, biological bandage, burn wound

## Abstract

Multidrug resistance infections are the main cause of failure in the pro-regenerative cell-mediated therapy of burn wounds. The collagen-based matrices for delivery of cells could be potential substrates to support bacterial growth and subsequent lysis of the collagen leading to a cell therapy loss. In this article, we report the development of a new generation of cell therapy formulations with the capacity to resist infections through the bactericidal effect of antimicrobial peptide dendrimers and the anti-virulence effect of anti-quorum sensing MvfR (PqsR) system compounds, which are incorporated into their formulation. Anti-quorum sensing compounds limit the pathogenicity and antibiotic tolerance of pathogenic bacteria involved in the burn wound infections, by inhibiting their virulence pathways. For the first time, we report a biological cell therapy dressing incorporating live progenitor cells, antimicrobial peptide dendrimers, and anti-MvfR compounds, which exhibit bactericidal and anti-virulence properties without compromising the viability of the progenitor cells.

## 1. Introduction

Loss of skin and immunosuppression following burns predispose patients to severe infections [1], with more than 50% of deaths following burn injury attributed to infections in the last decade [2]. The gold standard for the restoration of skin barrier after major burns is autografting of split thickness intact skin to cover the burned zones. However, in patients suffering from large surface burn wounds, intact skin for autografting is not available. Cultured epidermal autografts (CEAs), dermal regeneration templates (DRTs), and various forms of regenerative cell therapies and bioengineered skin constructs are the alternatives to autologous skin grafting to provide a permanent coverage of burn wounds and promote healing. Burn wounds are rapidly colonized by bacteria originating from the patient’s intact skin, gastrointestinal and respiratory flora, or external environment, with the latter group being more resistant to antimicrobial agents [3,4]. The transition of a colonized wound to an infected wound can happen in a permissive condition and is characterized by an increase of bacterial counts from the threshold of 10^5^ colony forming units (CFUs) per gram of tissue and the appearance of clinical signs of infection [5]. While the primary goal of the rapid and permanent coverage of burn wounds is to protect from infections, these regenerative cell-based therapies have occasionally been a ground for the initiation of infections in the clinic. In fact, infection of the DRTs and the graft site is a major early complication associated with this type of treatment [6,7,8].

Collagen, as the most abundant extracellular protein of the dermis, is the most widely used component of pro-regenerative DRTs and cell delivery matrices for the cell therapy of burn wounds [9,10,11,12]. On the other hand, together with laminin, collagen is a target for microbial adhesion, colonization and invasion of host tissue [13,14]. *Staphylococcus aureus* (*S. aureus*) and *Pseudomonas aeruginosa* (*P. aeruginosa*) are the most common bacteria causing infection of DRTs [6,15], and burn wounds in general [16]. *P. aeruginosa* produces endogenous proteases that degrade type I collagen [17,18,19] and indeed, increased bacterial growth in the presence of collagen membranes has been reported [20]. Thus, the presence of collagen can be a permissive factor for *P. aeruginosa*-initiated infections. This high risk of infection in patients treated with pro-regenerative cell therapies and DRTs implies the prescription of prophylactic pre- or post-operative antibiotics [21], increasing the risk of developing antibiotic resistance in patients. The emergence of multidrug resistant pathogens from burn infections is higher than other hospital-associated infections, which significantly limits the available therapeutic options for the effective treatment of burn wounds [1,22,23,24].

The development of antimicrobial scaffolds for burn wound regeneration is an active research domain, including dermal substitutes incorporating nano-carriers loaded with antibiotics for skin engineering [25] as an example. Replacement of classical antibiotics by non-antibiotic antimicrobial agents in such formulations is currently considered as a promising strategy to prevent infections and, at the same time minimizing the probability of inducing antibiotic resistance in burn patients. Non-antibiotic antimicrobials are substances that kill or stop the growth of bacteria, with a mechanism distinct from antibiotics. Unlike antibiotics that have specific mechanism of action by targeting distinct/single metabolic pathways, non-antibiotic antimicrobials have multiple molecular targets with non-specific mode of action [26]. Therefore, there is less risk of developing drug resistance through genetic mutations against these compounds [27].

Various non-antibiotic antibacterial compounds have been used to formulate scaffolds for cutaneous tissue regeneration. Under-development products such as cellulose-based biomaterial with mineral antimicrobials such as montmorillonite nanocomposites [28], chitosan-based antibacterial scaffolds [29,30,31] and burn dressings incorporating metallic antimicrobials such as silver nanoparticles [32,33,34] or other metallic antibacterial agents such as gallium ions [35] have shown promise in promoting wound healing while exhibiting antimicrobial properties.

Antimicrobial peptides (AMPs) are a key component of the innate immune system [36,37] with high antimicrobial effect but poor proteolytic stability. The structure of these molecules has been used as a model to develop synthetic analogues that mimic the properties of AMPs [38,39]. These synthetic AMPs, anti-microbial peptide dendrimers (AMPDs), exhibit increased activity due to their multivalent nature and resistance to proteases [38]. AMPDs represent potent alternatives to antibiotics [40,41], with quick and strong antimicrobial and antibiofilm activity against multiple Gram-positive and Gram-negative bacteria, including multidrug-resistant (MDR) microorganisms. There is no evidence of inducing resistance by AMPDs compared to conventional antibiotics [42,43,44].

Targeting bacterial virulence is an attractive strategy for combating MDR infections as it would disarm pathogens without affecting viability, thus reducing the selective pressures driving resistance [45,46]. Quorum sensing (QS) is a process of bacterial cell–cell communication through extracellular signaling molecules, to share information about population density and regulate their gene expression accordingly [47]. QS is a regulatory mechanism, crucial for the virulence, biofilm formation, and antibiotic resistance of many pathogenic bacteria including *P. aeruginosa* [48,49,50]. Disrupting QS may therefore be an effective strategy to combat recalcitrant infections. M59, a quorum sending inhibitor (QS-inhibitor) compound, binds to the global virulence QS transcriptional regulator MvfR (PqsR) and inhibits its activity, thereby blocks the transcription of a diverse array of virulence factors in multi-drug resistant isolates [51]. Disrupting QS and consequently bacterial virulence is therefore considered as an antimicrobial strategy combat recalcitrant infections [52].

**Figure 1:**
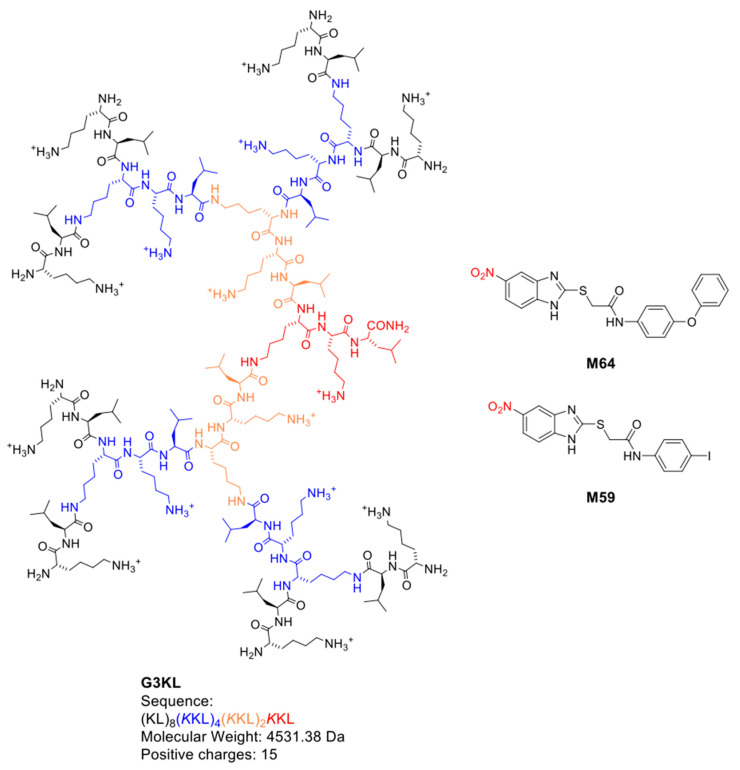
Chemical structure and sequence of antimicrobial peptide dendrimers (G3KL) and QS-inhibitor compounds (M59 and M64) tested in the present study. G3KL; third-generation dendrimer. First, second, and third generation residues are indicated in orange, blue, and black color, respectively. One letter codes for amino acids: (l, leucine; k, lysine). M95 and M64.

We have used pro-regenerative cell-based therapies consisting of clinical-grade progenitor skin fibroblasts for burns and wounds for more than two decades [10,11,53,54,55,56]. These cell therapy biological bandages are used as the first cover for the treatment of superficial to partial-thickness burns. The progenitor nature of these cells provides a rapid scarless healing efficiency with no immunological rejection, thereby substantiating the safety of application of the progenitor fibroblasts [12,57]. The first generation of these cell therapies is now in an ongoing clinical trial in Asia (trial ID numbers NCT03624023 and NCT02737748). In this article, we report the formulation of the second generation of these cell therapy dressings that, despite the usage of collagen as cell delivery matrix, is resistant to infection. Thus, this advanced cell therapy dressing will promote healing not only by providing the pro-regenerative factors delivered by progenitor skin fibroblasts, but also by having an antibacterial effect on colonized wounds and preventing the onset of infections. Since both wound colonization and infection hinder the process of wound healing, our advanced formulations are expected to have potentiated pro-regenerative effect on burn wounds. We used a dual targeting approach for our antibacterial strategy by incorporating in the collagen-based delivery matrix of the progenitor skin fibroblasts, two non-antibiotic antimicrobial compounds: the dendritic antibacterial peptide G3KL [58] and the QS-inhibitor M59 [51] (Figure 1).

## 2. Results and Discussion

### 2.1. G3KL Has a Rapid Onset of Bactericidal Effect and Is Active against Clinical Isolates of P. aeruginosa from Burn Wounds

**Figure 2:**
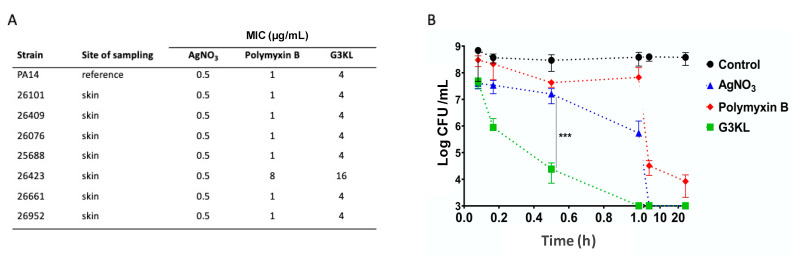
Antibacterial activity of G3KL. (**A**) Minimum inhibitory concentration of G3KL on clinical isolates and reference PA strain. (**B**) Time-kill assay of G3KL on PA14 reference strain. Silver nitrate and polymyxin B have been used as controls for bactericidal activity. Data are presented as the means ± SD (*n* = 3). *p* values were determined by unpaired Student’s test. *** *p* ≤ 0.001.

Multi-drug resistant *P. aeruginosa* infections are the most common causes of mortality in burn patients [1,16]. Thus, the antimicrobial compound to be incorporated in an ideal anti-infection burn dressing must be active against *P. aeruginosa* clinical isolates from burn wounds. We have recently synthesized a third-generation AMPD class, G3KL (Figure 1), which is active against *P. aeruginosa* and *A. baumannii* laboratory strains [58] as well as a broad panel of multidrug resistant clinical isolates of both of these pathogens [59], and also carbapenemase producing clinical strains [38,59]. G3KL has low hemolytic activity and exhibits excellent serum stability (half-life in human serum, [t1/2], of around 18 h), and its antimicrobial activity against *P. aeruginosa* is retained in the presence of human serum [58,60,61]. G3KL is active against both *P. aeruginosa* planktonic and biofilm cells at concentrations not toxic in vivo, which is comparable to Tobramycin [62]. The bactericidal effect of G3KL is a result of its interactions with the negatively charged lipids of the membrane, enabling its disruption with consequent rapid bacterial killing [58,63,64,65]. Of great clinical significance for burn treatment, we have shown that G3KL is active against polymyxin B resistant *P. aeruginosa* mutants and shows no detectable resistance selection induction [66]. Moreover, we have evidenced the proangiogenic effect of G3KL, which is crucial for the wound healing process [67]. We compared the bactericidal activity of G3KL on clinical isolates of *P. aeruginosa* from burn wounds by performing susceptibility assays on seven independent isolates from six burn patients and the PA14 as the reference burn wound isolate (Figure 2A). All strains showed similar minimum inhibitory concentrations (MICs) for silver nitrate, the active ingredient of commonly used antibacterial dressings applied on burn wounds. With the exception of isolate 26423, all isolates were also susceptible to polymyxin B, which was used for comparison of the bactericidal activity of G3KL. The MICs for G3KL were similar for all strains and in a similar range as for polymyxin B (1 µg/mL for polymyxin and 4 µg/mL for G3KL), with the exception of isolate 26423, which showed a four-fold decreased susceptibility to G3KL. Given the similar MICs for PA14 (reference burn wound isolate) and the burn wound isolates from our burn center, we used PA14 for the following experiments. Next, we assessed the kinetics of the bactericidal effect of G3KL on PA14 and compared the results with silver nitrate and polymyxin B, all compounds at concentrations corresponding to 4× their MICs. As shown in Figure 2B, polymyxin B caused a 5 logs reduction in PA14 CFUs/mL after 20 h incubation. Silver nitrate showed the rapid killing of PA14, with CFU counts dropping below our detection limit (10^3^ CFU/mL) within 3 h. G3KL displayed the most rapid killing kinetics, reducing CFU counts below the detection limit after less than 1 h of incubation. This rapid bactericidal effect is of great clinical significance in the treatment of burn wounds, mainly because almost all burn wounds are colonized at the time of the application of pro-regenerative biological dressings containing live cells. Therefore, rapid onset of the bactericidal effect is crucial to prevent the damage to the cells that are delivered to the wound bed by these dressings. Furthermore, since the delivery matrix for these cells is collagen-based, and collagen is specifically prone to degradation by proteases produced by *P. aeruginosa* [13,14,17,18,19], a rapid eradication of bacteria from the wound bed will preserve the viability and integrity of biological bandages.

### 2.2. Identifying the Cytocompatible Concentrations of AMPDs and QS-Inhibitors

The first generation of our cell therapy biological dressings was a combination of live progenitor skin fibroblasts that are delivered to the wound bed using a biodegradable collagen-based delivery matrix [10,12]. Our goal was to incorporate two classes of antimicrobial compounds (AMPDs and QS-inhibitors) into the delivery matrix in order to generate a novel generation of biological bandages with dual targeting of burn wound infections through contact killing and anti-virulence properties. We selected a third-generation AMPD, G3KL, with a strong bactericidal effect against *P. aeruginosa* which is the main pathogen causing multi drug resistance burn wound infections that also lead to chronic wound infections due the formation of biofilm and antibiotic tolerant cells that contribute to treatment failure. It is estimated that about 65% of all bacterial infections are associated with bacterial biofilms [68]. Biofilm formation is an important reason for the failure of antibiotic therapy and the development of resistance in bacteria [69]. The *P. aeruginosa* QS multiple virulence factor regulator (MvfR), controls many acute and chronic virulence functions, including the production pyoyanin and pyoverdine, and the formation of antibiotic tolerant cells and biofilm [50,70,71]. We have recently shown that burn wound exudate increases the production of pyocyanin and pyoverdin of *P. aeruginosa* [72]; therefore, anti-virulence compounds that target MvfR could have a major impact on the prevention and control of infections in burns [51]. For the formulation of our infection-resistant cell therapy dressings, we assessed the effect and cytocompatibility of two synthetic compounds with a potent inhibitory effect on the *P. aeruginosa* MvfR system; M59 and M64 (Figure 1) [51]. These two first-generation potent quorum sensing inhibitors that do not alter bacterial cell viability or growth, inhibit the *P. aeruginosa* virulence [51] by specifically binding and antagonizing MvfR [73]. As a result, they disrupt the MvfR-dependent cell-to-cell communication and MvfR-regulated virulence functions such as formation of antibiotic-tolerant bacteria and biofilms [74].

**Figure 3:**
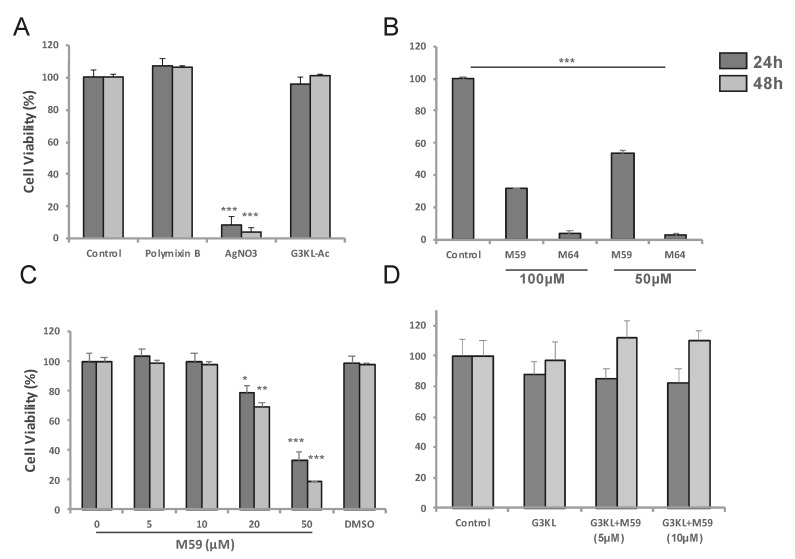
Assessment of the viability (MTT assay) of human progenitor fibroblast cells incubated with different antimicrobial compounds in 2D cultures. Cells were cultured for 24 to 48 h in the presence of: (**A**) acetate salt of G3KL as compared to antibiotic polymyxin B (12.5 µg/mL) and non-antibiotic antimicrobial silver nitrate (AgNO_3_; 6.25 µg/mL); (**B**) two QS-inhibitors (M59 and M64) at different concentrations; (**C**) lower M59 concentration; (**D**) G3KL in combination with M59. Bar charts show the percentage of viable cells quantified using the MTT assay, in comparison to the untreated live cells (100%). Data are presented as the means ± SD (*n* = 3). *p* values were determined by unpaired Student’s test. * *p* ≤ 0.05, ** *p* ≤ 0.01, *** *p* ≤ 0.001.

**Figure 4:**
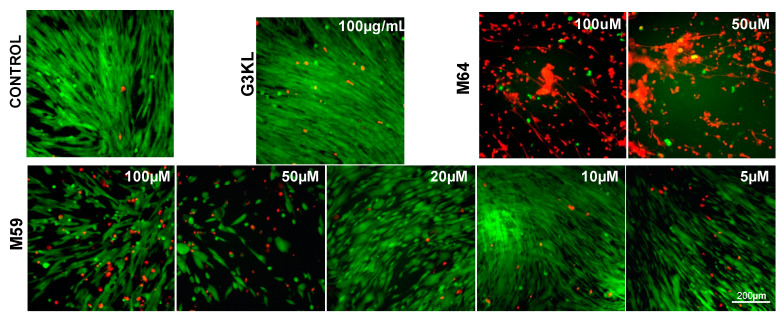
Assessment of the viability and morphology (Live/Dead assay) of human progenitor fibroblast cells incubated with different antimicrobial compounds. Cells were cultured for 24 h in the presence of G3KL or QS-inhibitors (M64 and M59). Higher concentration M64 and M59 induced death and morphological changes in cells. M59 did not induce cell death or morphology changes at concentrations below 20 μM. Live cells are stained green and dead cells are stained red.

Our selected antimicrobial compounds (G3KL, M59/M64) should preserve their activity after being incorporated into the collagen matrix in concentrations that do not affect the viability of the progenitor fibroblasts that are co-delivered with them onto the wound bed. First, we determined the cytocompatible concentrations of these compounds. The viability of progenitor skin fibroblasts incubated with AMPDs and QS-inhibitors for 24 and 48 h was assessed by measuring the mitochondrial activity of cells using an [3-(4,5-dimethylthiazol-2-yl)-2,5-diphenyltetrazolium bromide] (MTT) assay. We assessed the cytocompatibility of different analogs of G3KL (Supplementary Figure S1). We observed that the acetate salt of G3KL at 100 μg/mL did not affect the viability of cells at 24 and 48 h (Figure 3A), therefore, we selected the acetate salt of G3KL (G3KL hereafter) as the AMPD of choice for the formulation of antibacterial bandages. We also tested the cytocompatibility of our two QS-inhibitors, M64 and M59 [51]. M64 induced massive cell death in our progenitor fibroblast cultures, but M59 at concentrations below 20 μM did not alter the viability or morphology of progenitor fibroblasts (Figure 3B,C and Figure 4). M59 at 20 μM did not have strong toxicity on cells but induced a clear morphology change. We used polymyxin B and silver nitrate (AgNO_3_) as controls for anti-P. aeruginosa activity of the new formulations. AgNO_3_ had a strong toxic effect on the progenitor fibroblasts, but polymyxin B did not affect the viability of cells (Figure 3A). We also assessed the effect of a combination of non-cytotoxic concentrations of G3KL and M59 on the viability of progenitor fibroblasts. No cytotoxicity was observed when cells were treated with a combination of G3KL (100 μg/mL) and M59 (5 and 10 μM) (Figure 3D).

### 2.3. Identifying the Best Matrix for the Delivery of G3KL and Progenitor Skin Cells to Burn Wounds

**Figure 5:**
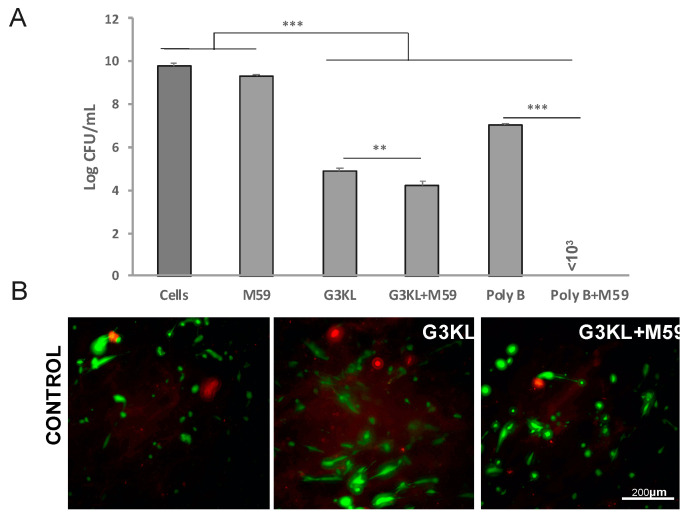
Assessment of the antibacterial effect and cytocompatibility of G3KL incorporated into a collagen-based solid matrix. (**A**) CFUs retrieved under the collagen matrix incorporating only progenitor cells, M59 (10 µM), G3KL (100 µg/mL), M59 (10 µM) and G3KL (100 µg/mL), polymyxin B (PolyB; 12.5 µg/mL), PolyB (12.5 µg/mL) and M59 (10 µM). (**B**) Live/Dead cell viability assay for progenitor cells seeded onto collagen matrix incorporating G3KL (100 µg/mL), or a combination of M59 (10 µM) and G3KL (100 µg/mL). Live cells are stained green and dead cells are stained red. Data are presented as the means ± SD (*n* = 3). *p* values were determined by unpaired Student’s test. ** *p* ≤ 0.01 *** *p* ≤ 0.001.

Microbes colonize all open wounds even in the absence of clinical signs of infection [75]. Therefore, any kind of wound dressing or cell therapies are applied on an already colonized microenvironment. Thus, an ideal wound dressing should exhibit both anti-infective and wound healing accelerating properties. G3KL, is an excellent candidate for preventing infections on wound sites. However, as AMPDs are a new class of antimicrobial compounds, there is no translational report on their delivery strategies for their application on wounds [76]. We use solid collagen-based delivery matrices for formulating the first generation of our proregenerative cell therapy dressings. Collagen is widely used in wound dressings and skin substitute products but is also susceptible to degradation by *P. aeruginosa*. For our new formulations, we sought to investigate other biopolymers as the delivery matrix. We assessed the bactericidal properties and cytocompatibility of G3KL at non-toxic concentrations after incorporation into two hydrogel formulations based on hyaluronic acid (HA; Ostenil) and chitosan (KitoKit) and compared the results with our collagen-based formulations. HA is a structural component of the extracellular matrix and is also widely used in tissues engineering and wound dressings [77,78]. HA derivatives are biodegradable and biocompatible and are involved in multiple biological functions, such as cell adhesion, migration, and proliferation, crucial to the wound healing process [79]. Chitosan is also a natural biomaterial with inherent antibacterial properties and is widely used in wound dressings. Chitosan promotes wound healing through its immune-modulatory effect and promotes fibroblast proliferation, migration, and ECM production [80,81]. G3KL retains its antibacterial activity after being incorporated into the collagen matrix and induces a 5 folds reduction in the bacterial load, retrieved from underneath the collagen-G3KL matrices (Figure 5A). The co-incorporation of M95 with G3KL potentiates the bactericidal effect of G3KL. Similar potentiating effect of M59 was observed for polymyxin B, which was used as a positive control (Figure 5A). In addition to the retention of their antibacterial effect after being incorporated into the collagen matrices, G3KL and M59 exhibited no toxicity to progenitor skin fibroblasts which were co-incorporated into the collagen matrix (Figure 5B).

**Figure 6:**
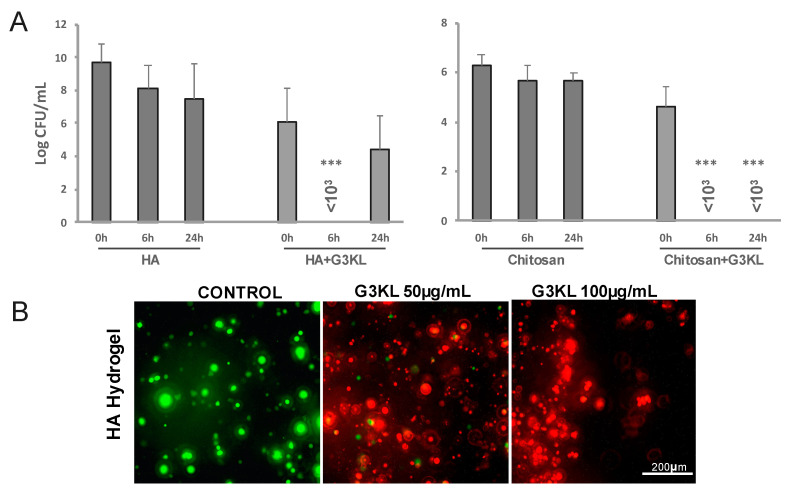
Assessment of the antibacterial effect and cytocompatibility of G3KL incorporated into HA and chitosan-based hydrogel matrices. (**A**) CFU count at different time points after incubation with hydrogels incorporating G3KL (100 µg/mL). (**B**) Live/Dead cell viability assay for progenitor cells seeded onto HA hydrogel incorporating G3KL (50 and 100 µg/mL). Live cells are stained green and dead cells are stained red. Data are presented as the means ± SD (*n* = 3). *p* values were determined by unpaired Student’s test. *** *p* ≤ 0.001.

Incorporation of G3KL into HA and chitosan did not abolish its antibacterial activity. HA-G3KL exhibited a rapid bacteriostatic activity with a 6-fold reduction in bacterial load after 24 h. G3KL-chitosan hydrogel showed a rapid and potent bactericidal effect (Figure 6A), but both hydrogel composites highly affected the viability of progenitor cells. As shown in Figure 6B and supplementary Figure S2, cells embedded in these hydrogels did not survive following 24 h incubation at 37 °C.

G3KL has a three-dimensional branched structure, and the charged end of the branches act as functional groups for their non-specific antibacterial effect. There are 15 positive charges in the structure of each G3KL molecule. The bacterial killing effect of G3KL is through penetration into the bacterial membrane and causing the leakage of cytoplasmic components leading to bacterial death [58]. We observed that fluorescently-labeled G3KL is uptaken by the progenitor skin fibroblasts in 2D cultures, with no effect on the viability of cells (Supplementary Figure S3). G3KL in the hydrated collagen matrix is not cytotoxic either; therefore, the configuration of the molecule in polymeric hydrogels might confer some toxic properties to G3KL. This toxicity is most probably independent of the charge of the polymer hydrogel as HA has overall negative charges, and chitosan is positively charged. Incorporation of G3KL in both turned to be cytotoxic. These experiments revealed that collagen-based solid scaffolds could be used as a cytocompatible delivery matrix for the G3KL (Figure 5B).

### 2.4. The Anti-Virulence Effect of QS-Inhibitor Compound M59 Is Retained When Embedded in the Collagen Matrix

**Figure 7:**
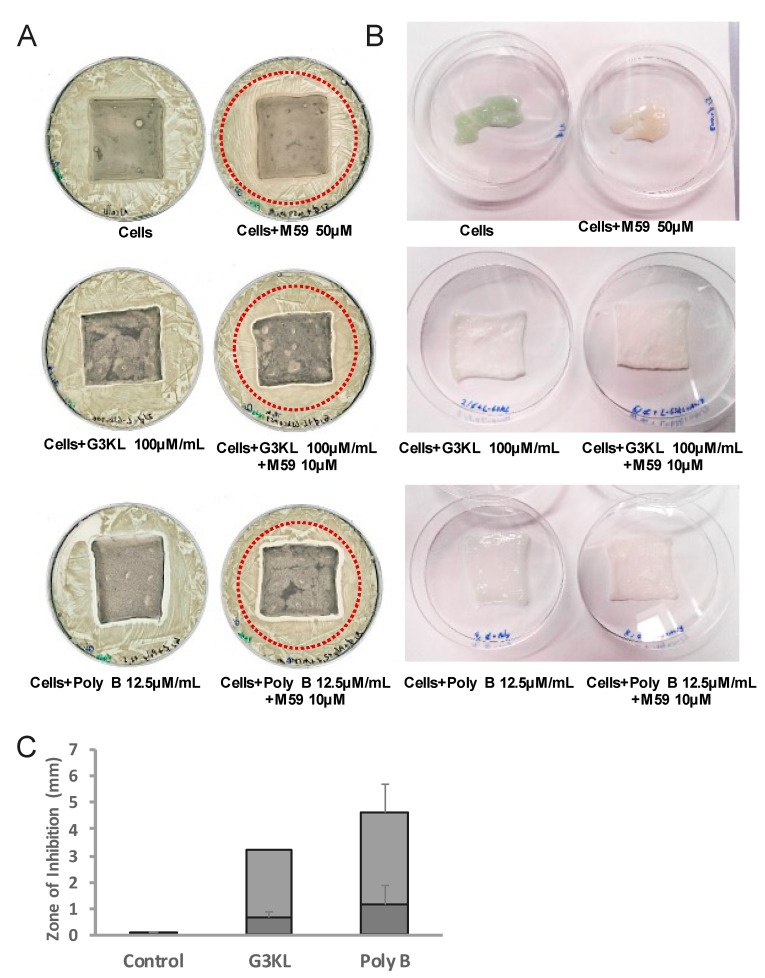
Antibacterial and anti-virulence properties of the final formulation of biological bandages on collagen matrix incorporating progenitor fibroblasts. The biological bandages were assembled with collagen matrix incorporating G3KL, M59, and progenitor fibroblasts. (**A**) The zone of growth inhibition surrounding the bandage (clear rectangle zone surrounding the matrix) shows the bactericidal effect of the dressing. The brown halo around dressings incorporating M59 (red dotted circle) shows the inhibition of pyocyanin expression. Only the formulating incorporating both G3KL and M59 shows both the inhibition zone and the inhibition of pyocyanin expression (absence of green color). (**B**) Images of matrices withdrawn from plates of panel (**A**). (**C**) Quantification of the zone of inhibition for bacterial growth. Dark grey: minimal inhibitory zone size, light grey: maximal inhibitory zone size.

M59 is a potent anti-virulence compound through the inhibition of the MvfR pathway in the QS system of *P. aeruginosa.* MvfR is a transcriptional regulator in *P. aeruginosa* that modulates the expression of several QS-regulated virulence factors [82] including pyocyanin [83]. Pyocyanin is a blue-green pigment and the green color of *P. aeruginosa* cultures is a result of the secretion of this virulence factor into the environment. M59 has a strong inhibitory effect on the production of pyocyanin through the inhibition of MvfR [51,84], thus we used the presence or absence of the green pyocyanin color as a read out for QS-inhibitory activity by M59 in our experiments. When incorporated into the collagen matrix, M59 with concentrations higher than 10 μM inhibited pyocyanin production, evidenced by the absence of green color from the matrix (Figure 7A,B, upper panel, and data not shown).

### 2.5. Scaleing up and Final Assembly of the Components of Novel Anti-Infection Cell Therapy Dressings

**Figure 8:**
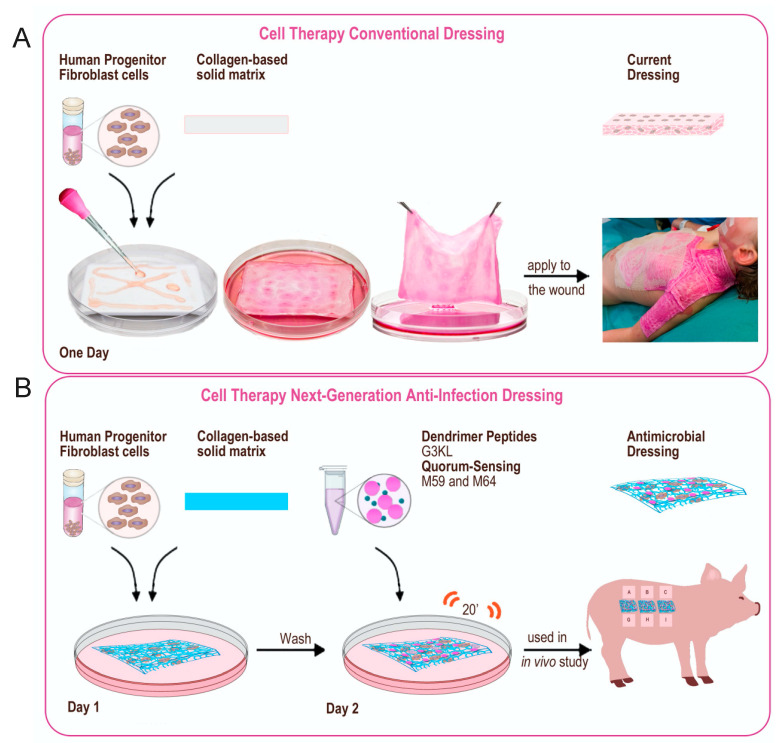
Final assembly of biological bandages. (**A**) first generation, (**B**) new anti-infection generation.

The process of the assembly of the first generation of our biological bandages for clinical use has been described previously ([12] and Figure 8A). Upon the admission of major burn patients in our Burn Unit at Lausanne University Hospital (CHUV, Lausanne) and if the patient meets the requirements to receive the progenitor cell therapy treatment (detailed in [12]), the number of required dressings to cover the burn wounds is estimated by the treating medical doctor, and the order is placed to the GMP cell production facility. Upon the reception of the production order, allogenic progenitor skin fibroblasts are withdrawn from the GMP clinical cell bank [85] and thawed for direct seeding on the solid collagen-based delivery matrix. Cells are washed after thawing and resuspended in a freshly prepared complete DMEM medium. The viability of cells is determined and if more than 80% viable cells are available, the batch is released for the construction of the biological bandages. Cells are diluted to 10^5^ total cells/mL and homogenously seeded on preconditioned collagen scaffolds. The constructs (biological bandages) will be incubated for 24 h at 37 °C before multiple washes and transfer to the operating room for clinical application on burn wounds ([12] and Figure 8A).

Following the same procedure for incorporating cells into the collagen delivery matrix, we explored multiple scenarios for embedding the antibacterial components into the biological bandages for our new formulations. We assessed the antibacterial activity of fully assembled next-generation dressings after suspending the M59 and G3KL at cytocompatible concentrations (10 μM and 100 μg/mL respectively) in pre-incubation wash solutions, overnight incubation solution, post-overnight incubation wash solutions. The suspension of antibacterial compounds in the overnight incubation solution yielded good incorporation of compounds into the construct and the retention of activity, evidenced by the formation of growth inhibition zone and blocking the expression of pyocyanin (Figure 7B and data not shown). However, to achieve the desired concentrations of the M59 and G3KL, large amounts of compounds were needed. Therefore, we tried a 20 min incubation (soaking under gentle agitation) of constructs in PBS containing M59 and G3KL after the overnight incubation and wash processes at the last step of the assembly. We also determined the minimum soaking volume and found that a minimum of 0.5 mL soaking suspension per cm^2^ of collagen matrix surface is needed to obtain adequate incorporation of antibacterial compounds into the delivery matrix. We assembled the new anti-infection cell therapy dressings under the new established protocol and assessed their antibacterial efficacy against the PA14 clinical strain (Figure 7A,B). As predicted, the collagen scaffolds devoid of G3KL, got digested by the activity of proteinase enzymes of *P. aeruginosa*. As shown in Figure 7B (upper panel), the digested matrix had lost its integrity upon retrieval from the surface of the PA14 solid culture. The presence of M59 alone did not prevent the infection and degradation of the collagen matrix but prevented the production of pyocyanin, as evidenced by the absence of green color as compared to control collagen matrix (Figure 7B, upper panel). In contrast, the incorporation of G3KL prevented the infection and degradation of the dressings, as the intact matrix could be retrieved from the PA14 culture (Figure 7B, middle panel). The bacterial growth inhibition zone around the dressings containing G3KL and the absence of green color shows the anti-infective properties of the new generation of dressings. The antibacterial activity is comparable with polymyxin B incorporated dressings (Figure 7A,C).

Multiple repetitions of these experiments confirmed that the explained process for constructing the new anti-infection dressings (Figure 8B) is effective and efficiently prevents the infection of the collagen-based cell therapy scaffold and has a contact-killing and anti-virulence effect on the *P. aeruginosa* clinical isolate, PA14. Taken together, using a stepwise approach, we designed a second generation of pro-regenerative cell-mediated therapies that successfully deliver within the burn wound anti-bacterial as well as anti-virulence mediators. The final formulations of theses dressings are currently, under investigation in vivo, in minipig model of burn wound infection and we expect them to reach the bedside in the next years.

## 3. Materials and Methods

### 3.1. Bacterial Strains, Clinical Grade Human Fibroblast Cells, and Chemicals

*P. aeruginosa* PA14 strain was kindly provided by the laboratory of Prof. L G Rahme, University of Harvard and used as a reference strain. The clinical burn wound isolates were collected from patients hospitalized between 2012–2014 at the CHUV (kindly provided by D. Blanc, CHUV). If not otherwise stated, the bacterial strain was cultured in LB medium at 37 °C with shaking (250 rpm). The Antimicrobial Peptide Dendrimers (AMPDs) were provided by the University of Bern (Prof. JL Reymond; [58]. The quorum sensing inhibitors (M59 and M64) were provided by the University of Harvard (Prof. L G Rahme; [51]). Silver nitrate (AgNO_3_) and polymyxin B were purchased from SIGMA-Aldrich (Buchs, Switzerland). Human skin progenitor fibroblasts were obtained from a registered primary cell bank (FE002-SK2, used for experimentation between passage 2 and 6) isolated from an organ donation according to a protocol approved by the State ethics committee (University Hospital of Lausanne, Ethics Committee Protocol # 62/07) and within regulations of the Department Biobank. These cells were personally donated for specific research purposes by Prof. Applegate.

### 3.2. Cellular Toxicity Assays for Antibacterial Compounds

#### 3.2.1. Live/Dead Assay

Progenitor skin fibroblasts were grown within 96-well microplates (DMEM medium complemented with 10% FBS, 1% glutamine) in a standard incubator (37 °C, 5% CO_2_) for 24 h. The culture medium was then replaced with a medium containing different antimicrobial compounds, and cells were further incubated at 37 °C for 24 h. After incubation, the culture medium was removed, washed twice with PBS, and the viability was assessed using the Viability/Cytotoxicity Assay Kit for Animal Live and Dead Cells (Biotium, Hayward, CA, USA). Reagent solutions were prepared according to manufacturer protocol and added to cultures and incubated for 45 min at room temperature. Stained cells were observed, and images were taken using a fluorescent microscope (Zeiss Axiovert 100, Oberkochen, Germany).

#### 3.2.2. MTT Cell Titer Assay

Progenitor skin cells were grown and incubated with antimicrobial compounds as described in the previous section. After 24 h of incubation, cell viability was measured using the CellTiter 96^®^AQueous One Solution Cell Proliferation Assay Kit (Promega, Madison, WI, USA). Reagent solution was prepared in DMEM culture medium according to manufacturer’s protocol. 150 μL of the medium was added to each culture and incubated for 90 min. Further, 100 μL of medium was transferred to a fresh 96-well microplate, and the absorbance was measured at 490 nm with a spectrophotometer (Wallac Victor2 1420 multilabel counter, PerkinElmer, MA, USA).

### 3.3. Preparation of Cell-Embedded Dressings

*Dressings with solid collagen matrix:* Biological bandages were prepared as described previously [10,12]. In brief, frozen fetal progenitor skin fibroblasts (passage 2) from the Clinical Master Cell Bank [86] were thawed, washed twice with PBS, and re-suspended in completed DMEM. Collagen TissueFleece™ (Baxter, Heidelberg, Germany) delivery matrix was cut into 4 × 4.5 cm square sections. Cells were evenly seeded into the collagen matrix (5000 cells/cm^2^) and incubated at room temperature for 20 min, the time necessary for cells to adhere to the matrix. The biological bandages were incubated overnight at 37 °C, followed by multiple washes with PBS. Antimicrobial peptide dendrimers and QSIs were suspended in PBS and added to the cell-seeded matrices, soaked for another 20 min and used in the antibacterial activity assays. Control bandages did not contain antibacterial compound.

### 3.4. Assessment of Antibacterial Activity

#### 3.4.1. Minimal Inhibitory Concentrations (MIC) Assessment

The broth microdilution method was used to determine the MIC of antimicrobial compounds against PA14 strain, according to Clinical Laboratory Standards Institute guidelines [87]. A single bacterial colony was grown in MHB (Mueller-Hinton Broth) medium overnight at 37 °C. The stock solutions of antimicrobial compounds were prepared and added to the first well of 96-well microtiter plate and two-fold dilutions were made. Bacteria was added to each well to a final inoculation of about of 5 × 10^5^ CFUs. The plates were incubated at 37 °C for 18 h. For each test, two columns of the plate were kept for sterility control (broth only) and growth control (broth with bacterial inoculums, no antibacterials). The MIC was defined as the lowest concentration of the peptide dendrimer that inhibited visible growth of the PA14. Following MIC reading, the lowest concentration of the test agent killing at least 99.99% of the original inoculum was measured by plating 100 μL broth from clear wells on MHA plates and incubation at 37 °C for 24 h for CFU count.

#### 3.4.2. Inhibition Zone Assay

The antibacterial activity of cell-embedded dressings against Gram-negative *P. aeruginosa* was tested using the inhibition zone assay. Solid agar plates containing M9 salt medium supplemented with 0.2% glucose, 0.5% casamino acids (CAA), 2 mM MgSO_4_ and 1.6% agar, which were prepared 24 h in advance. To inoculate the agar plate, 100 μL of NaCl 0.9% containing 10^8^ CFU/mL were spread on the plate using a sterile rake and dried under a laminar flow for 15 min. Cell-embedded dressings were prepared, and AMPDs were incorporated into the dressings as described earlier and were placed on *P. aeruginosa* inoculated agar plate by means of sterile tweezers and incubated at 30 ^◦^C for 18 h. The positive control consisted of the dressing incorporating polymyxin B (4 mg/L), and negative controls did not incorporate any antibacterial compound. The next day the plate was scanned, and inhibition zones were measured. Matrices were removed from the plate and photographed. The experiments were carried out in triplicate in two experimental replicates.

#### 3.4.3. Bacterial Killing Assay

Bacterial PA14 suspensions were prepared by resuspending culture grown overnight in LB medium and washed once in M9 salts supplemented with 2 mM MgSO_4_ (M9MS), at an OD_600_ = 2.0 (5 × 10^9^ CFU/mL). 15 uL of the above cell suspension was diluted in 96-well plates containing 135 uL of M9MS and 4× the MIC of the test compound (2 mg/L AgNO_3_, 4 mg/L polymyxin B and 32 mg/L G3KL) and incubated at 37 °C. Samples were withdrawn at regular time points and serially diluted in 0.9% NaCl. Five uL of appropriate dilutions were plated on LB agar plates and incubated for 18 h at 37 °C. The number of colony forming units (CFU) was counted at the end of incubation. Experiments were performed in triplicate and on three separate occasions.

#### 3.4.4. Antibacterial Properties of Hydrogel Formulations

A total of 10^8^ bacteria were suspended in 200 µL of the gel formulations (HA or Chitosan) in a 96-well plate and incubated at 30 °C. At each time point, CFU count was performed by removing 10 µL aliquots and preparing 10-fold serial dilutions before spotting samples on LB-agar plate.

## 4. Conclusions

As all open wounds may be colonized with microbes, even in the absence of clinical signs of infection [75], any kind of wound dressing or cell therapies is applied to an already colonized microenvironment. The likelihood that the microbial colonization of a wound leads to infection depends on both host and microbial factors. Therefore, an ideal wound dressing should exhibit both anti-infective and wound-healing accelerating properties. Integration of antibiotics into wound regeneration products would prevent infection; however, the risk of antibiotic resistance and inherent cytotoxicity of some antibiotics [88] decreases the enthusiasm towards their application in wound regenerative therapy. Our study shows for the first time the possibility of combining wound healing properties provided by progenitor cells with antimicrobial and anti-virulence agents in a burn wound cell therapy dressing. Moreover, we also report for the first time a translational study to identify the best delivery system for AMPDs on wound sites, paving the way to use them in a clinical setup.

## Data Availability

The data presented in this study are available on request from the corresponding author.

## Supplementary Materials

The following are available online, Figure S1: Assessment of the viability (MTT assay) of human progenitor fibroblast cells incubated with different analogs of G3KL. Figure S2: Assessment of cytocompatibility of G3KL incorporated into Chitosan-based hydrogel matrix. Figure S3: Cytotoxicity and cellular uptake of Fluorescent-G3KL.

## Sample Availability

Not available.
