# Supplementary material for: Antimicrobial Peptide Dendrimers and Quorum-Sensing Inhibitors in Formulating Next-Generation Anti-Infection Cell Therapy Dressings for Burns
Source: Molecules. 2021 Jun 24;26(13):3839. doi: 10.3390/molecules26133839 (PMC8270311; doi:10.3390/molecules26133839)
Supplement: Supplementary file 1 [file molecules-26-03839-s001.zip › molecules-1227466-supplementary.pdf]

Supplementary Figures.

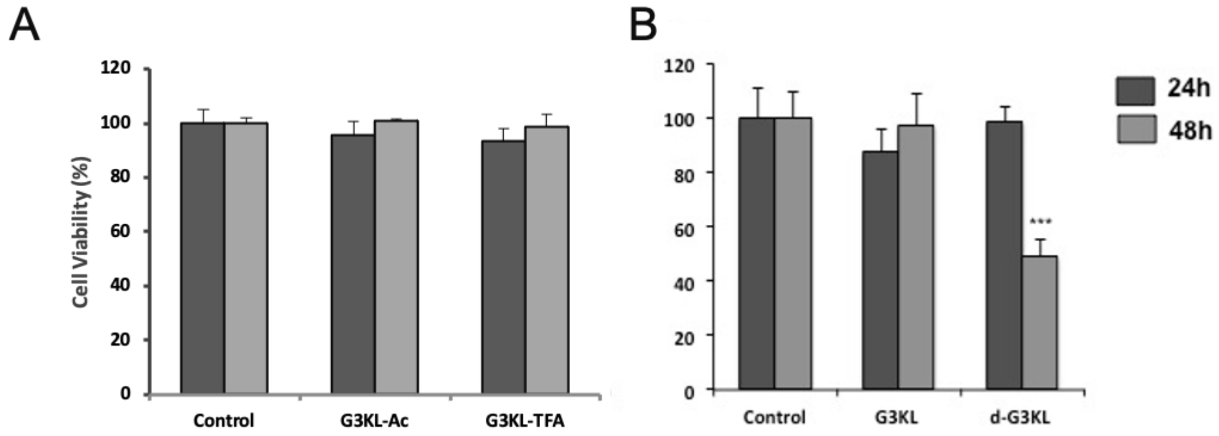

Supplementary Figure S1. Assessment of the viability (MTT assay) of human progenitor fibroblast cells incubated with different analogs of G3KL. Cells were cultured for 24 to 48 hours in the presence of: A) acetate salt and TFA salt of G3KL. B) two stereoisomers of G3KL. Bar chart shows the percentage of viable cells quantified using the MTT assay. Values are shown in comparison to the untreated live cells (100%). Data are presented as the means ± SD (n = 3). P values were determined unpaired Student's test. ***P ≤ 0.001

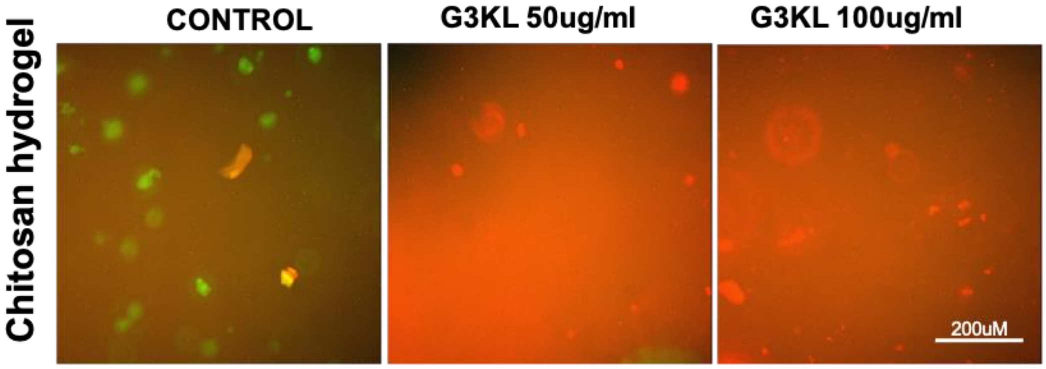

Supplementary Figure S2. Assessment of cytocompatibility of G3KL incorporated into Chitosan-based hydrogel matrice. Live/Dead cell viability assay of progenitor fibroblast cells incorporated into Chitosan hydrogel and Chitosan hydrogel incorporating different concentrations of G3KL (50 and 100 μg/ml). Live cells are stained green and dead cells are stained red.

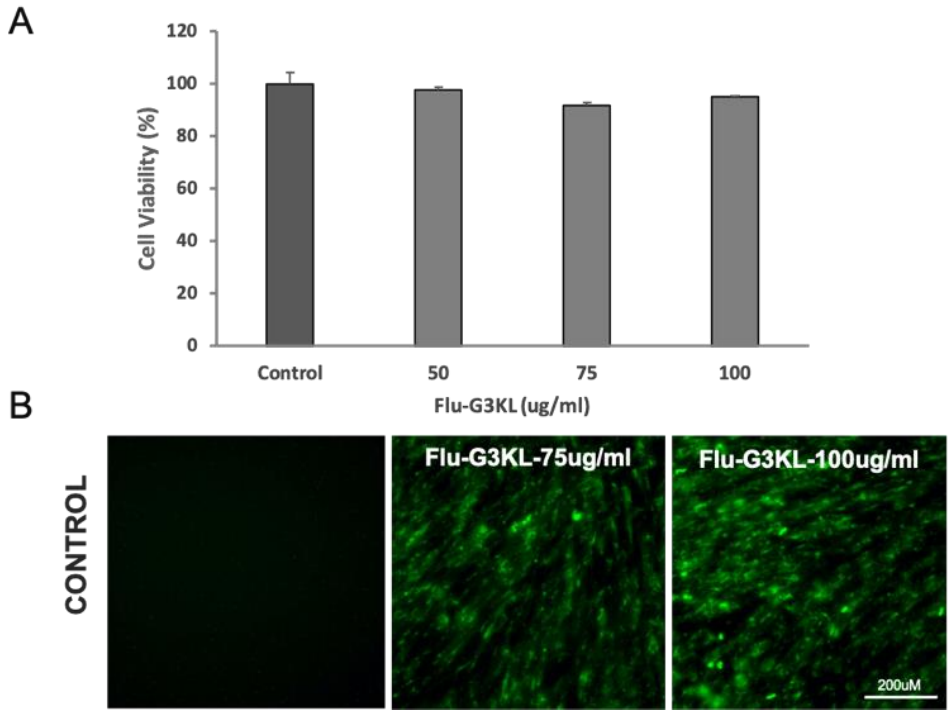

Supplementary Figure S3. Cytotoxicity and cellular uptake of Fluorescent-G3KL. A) Viability of progenitor fibroblasts incubated with different concentrations of G3KL labeled with Fluorescein (Flu-G3KL), measure by MTT assay. Data are shown in comparison to the untreated live cells (100%). B) Flu-G3KL uptakes by progenitor fibroblasts cells after 24 hours of incubation with culture media containing different concentrations of Flu-G3KL. Values are expressed as mean ± SD.
